# A journal for global health programming

**DOI:** 10.9745/GHSP-D-13-00001

**Published:** 2013-03-21

**Authors:** James D Shelton, Ronald J Waldman

**Affiliations:** a Co-Editor-in-Chief, Global Health: Science and Practice

## Abstract

GHSP aims to improve how programs function at scale, targeting implementers who actually support and carry out programs across all of global health. Thus, we emphasize specific implementation details, using a crisp, accessible, interactive style.

What motivated us to initiate *Global Health: Science and Practice* (GHSP)?

## MOMENTOUS GLOBAL HEALTH AGENDA

Infectious diseases such as pneumonia, HIV, malaria, and tuberculosis continue to plague much of the world. Reproductive health issues including unintended pregnancy and maternal mortality are stubbornly persistent. The Child Survival Call to Action, recently announced by the Governments of Ethiopia, India, and the United States with UNICEF, calls for ending preventable child death by 2035. And the Global Burden of Disease Study 2010 highlighted the ever-growing burden of non-communicable disease and injury looming large on the horizon. To rise to the challenges, we must understand the best approaches and implement the best programs.

## PROGRAMMING AT SCALE, REQUIRING BOTH FORMAL RESEARCH AND EXPERIENTIAL KNOWLEDGE

Many journals focus on clinical issues and carefully controlled research. We also will publish such research across a wide range of methodologies—from randomized controlled trials to field-level observational studies. But working at scale is essential to meeting our huge global health challenges. And mounting a successful program to address health issues at large scale entails a level of complexity and requires appropriate adaptation to specific local situations involving a host of implementation details. Formal research definitely helps. But it is not enough. We also need programmatic know-how, including experience-based knowledge and “lessons learned.” We aim to draw on a wide variety of relevant disciplines, such as evaluation, management science, behavior change, political science, and engineering. The key challenge for applying experiential knowledge is to find principles and lessons learned that are systematic, replicable, and applicable in other settings.

## A JOURNAL FOR PROGRAM IMPLEMENTERS

For programmatic knowledge to have value, it must be used by those actually involved in designing, implementing, and supporting programs on the ground, especially in low- and middle-income countries. Would such a person find useful knowledge and “lessons learned” in an article that they could apply to their own programs? We strive to ensure that program implementers find such practical know-how in GHSP. Accordingly, while we are interested in whether a particular intervention is successful, we encourage authors—many of whom are program implementers on the ground themselves—**to provide a high level of detail on how activities were actually conducted—the kind of implementation detail most other journals tend to shy away from.**

## BETTER INTERACTIVITY AMONG GLOBAL HEALTH COMMUNITY OF PARTNERS

As global health advances, engagement has increased from donors, NGOs, commercial sector partners, and even consortia of such partners. Moreover, governments, civil society, and consumers are assuming greater and greater roles. Improved technology allows for a more accessible, inclusive approach that provides potential to connect with many more colleagues around the world. And our aim is to be as interactive as possible, by allowing readers to submit formal letters to the editor, but also by posting comments on articles and engaging in discussions through social media, including Facebook and Twitter.

## OPEN-ACCESS PUBLISHING FOR BOTH READERS AND AUTHORS

We firmly believe that reducing barriers to accessing health information can speed up the pace of scientific discovery, encourage innovation, and, in many low- and middle-income countries, can even mean the difference between life and death. Thus, GHSP supports the open-access movement by making articles freely available to read, use, and distribute (original author and source should be properly cited) and also by not charging authors article-processing fees to submit and publish their work with GHSP.

## CRISP, EFFICIENT COMMUNICATION STYLE THAT TACKLES KEY ISSUES

People implementing programs are busy implementing. And others in the global health community also need efficiently packaged and thought-stimulating literature to meet their needs. So we will aim for such a crisp style, one that lays out key concepts prominently. Each article will be accompanied by a SOCO—a single overriding communication objective. And we aim to address those global health issues that have the most programmatic importance, global policy relevance, and potential impact, including those that may engender controversy in an engaging and sometimes provocative way. Thus, much of our space will be devoted to commentaries, syntheses, and even debates about key global health developments and issues.

A new communicative, open-source, peer-reviewed journal, from a source considered trusted, is but one of many tools needed to reach the ambitious goals of global health for the next generation. But GHSP's success will depend, in no small measure, on your participation—by authoring, by reviewing, by reading and sharing, and by commenting—but most importantly, by doing.

